# Improved segmentation accuracy in high-resolution peripheral quantitative computed tomography scans of carpal bones using adaptive local thresholding

**DOI:** 10.1093/jbmrpl/ziag054

**Published:** 2026-03-30

**Authors:** Michael T Kuczynski, Simone Poncioni, Sarah Elmahdy, Erica Baldesarra, Philippe Zysset, Sarah L Manske, Danielle E Whittier

**Affiliations:** McCaig Institute for Bone and Joint Health, University of Calgary, Calgary, AB, Canada; Department of Cell Biology and Anatomy, Cumming School of Medicine, University of Calgary, Calgary, AB, Canada; Department of Radiology, Cumming School of Medicine, University of Calgary, Calgary, AB, Canada; ARTORG Center for Biomedical Engineering Research, University of Bern, Bern, Switzerland; McCaig Institute for Bone and Joint Health, University of Calgary, Calgary, AB, Canada; Department of Cell Biology and Anatomy, Cumming School of Medicine, University of Calgary, Calgary, AB, Canada; Department of Radiology, Cumming School of Medicine, University of Calgary, Calgary, AB, Canada; McCaig Institute for Bone and Joint Health, University of Calgary, Calgary, AB, Canada; Department of Cell Biology and Anatomy, Cumming School of Medicine, University of Calgary, Calgary, AB, Canada; Department of Radiology, Cumming School of Medicine, University of Calgary, Calgary, AB, Canada; ARTORG Center for Biomedical Engineering Research, University of Bern, Bern, Switzerland; McCaig Institute for Bone and Joint Health, University of Calgary, Calgary, AB, Canada; Department of Radiology, Cumming School of Medicine, University of Calgary, Calgary, AB, Canada; Department of Biomedical Engineering, Schulich School of Engineering, University of Calgary, Calgary, AB, Canada; McCaig Institute for Bone and Joint Health, University of Calgary, Calgary, AB, Canada; Department of Cell Biology and Anatomy, Cumming School of Medicine, University of Calgary, Calgary, AB, Canada; Department of Radiology, Cumming School of Medicine, University of Calgary, Calgary, AB, Canada

**Keywords:** HR-pQCT, trabecular bone, segmentation, bone microarchitecture, carpal bones

## Abstract

The choice of segmentation method for HR-pQCT scans influences accuracy of bone microarchitecture measurements. Smaller or under-mineralized bone can present challenges in accurate extraction of bone structure using global thresholding methods, as local variations in intensity are not considered and finer structural details are not detected. This is especially important in small hand bones, where bone structure is finer, or younger populations, where bone tissue may be under mineralized. This study compared accuracy of global thresholding methods using Gaussian and Laplace–Hamming filters, and an adaptive local thresholding (AT) method in HR-pQCT scans of carpal bones. Eight ex vivo human cadaveric forearms (*n* = 64 carpal bones) were analyzed. Three specimens (*n* = 24 carpal bones, 2 female, mean age: 82.7 ± 4.6 yr) were used for AT parameter optimization, and 5 specimens (*n* = 40 carpal bones, 3 female, mean age: 82.0 ± 6.4 yr) were used to compare trabecular microarchitecture accuracy and spatial agreement relative to micro-CT (μCT, 20 μm isotropic resolution). Micro-CT images were segmented using a Gaussian filter and Otsu’s method, and HR-pQCT images were segmented using Gaussian filtering and global thresholding, Laplace–Hamming filtering and global thresholding, and the AT method. Trabecular thickness (Tb.Th), separation (Tb.Sp), and bone volume fraction (Tb.BV/TV) accuracy were evaluated, and spatial agreement was assessed using Dice similarity coefficients (DSC), 95th percentile Hausdorff distances (HD95), and average symmetric surface distances (ASSD). The AT method yielded the smallest absolute and relative errors, and lowest bias across all trabecular parameters. Compared to the Gaussian and fixed threshold method, AT reduced mean absolute error by 36% for Tb.Th, 14% for Tb.Sp, and 15% for Tb.BV/TV, and achieved the highest spatial agreement with μCT (DSC = 0.84, HD95 = 0.061 mm, and ASSD = 0.018 mm). These findings extend prior HR-pQCT segmentation validation studies to carpal bones and demonstrate that AT outperforms the standard and Laplace–Hamming methods.

## Introduction

HR-pQCT allows for 3-dimensional in vivo assessment of bone microarchitecture of peripheral skeletal sites, most commonly in the distal radius and tibia. However, application to other peripheral sites, such as small joints of the hand have begun to emerge and show promise for studying the effects of degenerative conditions or injury in exceptional detail.^1–8^ HR-pQCT image processing pipelines that primarily utilize the scanner manufacturer’s scripting language have been validated for the distal radius and tibia^9,10^ but not for the small bones of the hand. The carpal bones pose a unique challenge for HR-pQCT segmentation due to their irregular shape, numerous articulations, and thin cortical shell, making accurate classification of trabecular bone challenging.

In second-generation HR-pQCT (XtremeCT II, 61 μm isotropic voxels), the standard bone segmentation method uses a Gaussian smoothing filter, followed by separate fixed thresholds for cortical and trabecular bone compartments.^11^ Recently, a Laplace–Hamming filter was shown to improve bone segmentation accuracy of second-generation HR-pQCT scans of the distal radius and tibia compared to the standard Gaussian approach, for cases where finer structural features are present.^12^ Motion artifacts and local variations in bone mineralization can cause nonuniform intensity distributions, making global threshold-based segmentation methods prone to over- or under-segmentation. The standard HR-pQCT workflows that have been validated for the distal radius and tibia may not accurately extract bone structure in smaller or under-mineralized bones.

Alternatively, adaptive local thresholding (AT) methods have been proposed to improve bone segmentations in high-resolution CT imaging.^13–15^ These methods compute a threshold for each voxel using the intensity range of neighboring voxels. In first-generation HR-pQCT (XtremeCT, 82 μm isotropic voxels), Burghardt et al. demonstrated that AT improved trabecular segmentation in femoral trabecular bone samples compared to global thresholding.^13^ Mys et al. extended these findings to second-generation HR-pQCT scans of the distal radius, demonstrating that AT improved accuracy of trabecular and cortical bone segmentations.^14^ These studies demonstrate improved segmentation accuracy in long bones, however, their performance in smaller carpal bones has not been established and may require further optimization.

The purpose of this study was to evaluate segmentation accuracy in HR-pQCT scans of carpal bones using the (1) standard Gaussian, (2) Laplace–Hamming, and (3) AT methods. A parameter optimization was performed to determine AT parameters for carpal bones. Using global threshold on micro-CT (μCT) as a gold-standard, we assessed spatial overlap and accuracy of trabecular bone microarchitecture between HR-pQCT segmentation methods.

## Materials and methods

### Image data

We analyzed publicly available, previously acquired HR-pQCT and μCT scans of 8 cadaveric human forearms from the Multimodal CT Dataset of Cadaveric Wrist Joints.^16^ The HR-pQCT scan (XtremeCT II, Scanco Medical; protocol: 68 kV voltage, 1470 μA tube current, 100 ms integration time, and 60.7 μm isotropic voxel resolution) acquisition began at the distal radius and extended 81.6 mm distally (1344 axial slices or 8 sequential image “stacks”). HR-pQCT image gray values were calibrated to hydroxyapatite (HA) density using the scanner’s standard asynchronous calibration protocol, and all images were converted to density units (mg HA/cm^3^) prior to further processing. All HR-pQCT scans were acquired on the same day using identical acquisition parameters, minimizing potential scan-to-scan variability. Following this, the individual carpal bones were extracted from each specimen (*n* = 64 total) and were scanned with μCT (Bruker Skyscan 1272; protocol: 100 kV voltage, 100 μA tube current, 2200 ms exposure time, and 20 μm isotropic voxel size). From this dataset, three specimens (*n* = 24 carpal bones) were used for AT parameter optimization for carpal bones, and the remaining 5 specimens (*n* = 40 carpal bones) were used to assess accuracy of the standard, Laplace–Hamming, and AT methods against μCT.

### Image processing and analysis

**Figure 1:**
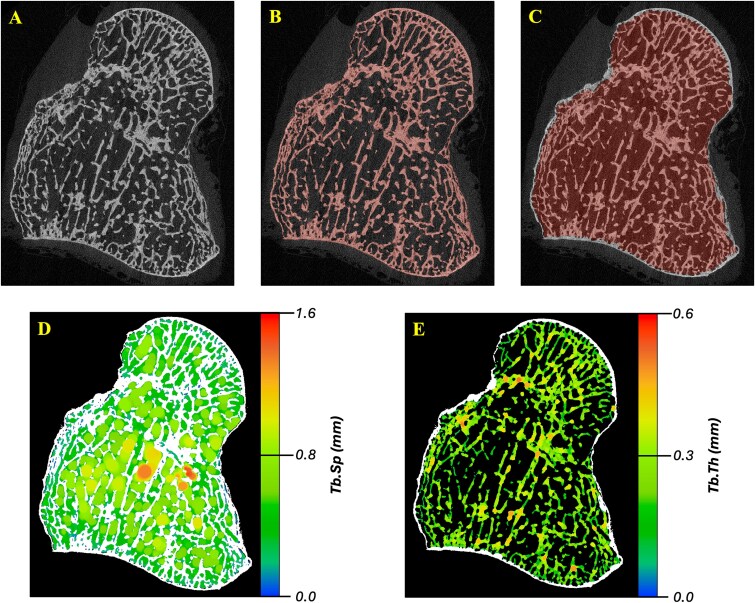
Micro-CT image processing workflow. The original μCT image (A) is segmented using a Gaussian smoothing filter and Otsu’s method to select a global threshold for bone (B). A trabecular volume of interest is defined (C), and trabecular separation (D) and thickness (E) are computed within the volume of interest using a sphere filling method.

The μCT images were segmented using a Gaussian smoothing filter (σ = 2.0, support = 1.0) and Otsu’s method to determine a scan-specific global threshold for each μCT image (Figure 1B). A trabecular volume of interest (VOI) was defined following a previously described method^17^ (Figure 1C). Each trabecular VOI was manually assessed and corrected using ITK-SNAP^18^ (v4.0.2).

**Figure 2:**
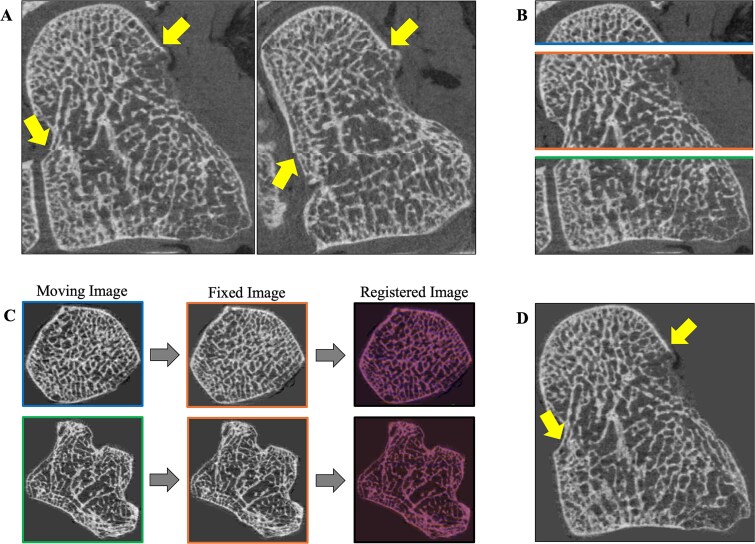
HR-pQCT image processing steps for the stack-shift artifact correction. Each HR-pQCT image was manually inspected for stack-shift artifacts (A). If present, individual stacks were manually cropped using ITK-SNAP (B), and 2D slices from neighboring stacks were registered to minimize the stack-shift artifact (C). The resulting 2D transform was applied to the 3D image stacks to form the correct image (D).

Carpal bones were manually cropped from HR-pQCT scans using ITK-SNAP and assessed for inherent stack-shift artifacts that arise due to the image acquisition approach. We corrected stack-shift artifacts in line with methods reported by Bevers et al., by extracting 2D axial slices from the stack boundaries and performing a rigid 2D registration between adjacent slices to determine an in-plane translation and rotation^19^ (metric: normalized correlation, interpolator: B-Spline, optimizer: gradient decent, and using a multi-resolution framework). The resulting 2D transform was applied to the 3D stack images to correct the stack-shift artifact (Figure 2).

Using the autocontour module from the ORMIR_XCT package (v.1.0.3), a periosteal bone mask was generated for HR-pQCT scans of each carpal bone and used to isolate each carpal from surround bones. Next, μCT images were rigidly registered to their corresponding HR-pQCT image using Mattes Mutual Information, a linear interpolator, gradient decent optimizer, and multi-resolution framework. The resulting transformation matrix was used to transform the μCT VOI mask and bone segmentation to the HR-pQCT image space (interpolator: nearest neighbor).

To determine optimal parameters for the AT, we performed a parametric analysis that captured previously described parameters implemented for the distal radius (Gaussian filter, spherical kernel with radius of 3 voxels, and lower threshold of 240 mg HA/cm^3^).^14^ Thus, we iterated over kernel radius (3, 6, and 9 voxels), minimum structure size (16, 32, and 64 voxels), and lower threshold (250-500 mg HA/cm^3^ in increments of 5 mg HA/cm^3^). The minimum structure size determined the 3D connected-component threshold, which eliminated any connected components smaller than this value. For each iteration, a spherical kernel was used, and the local threshold at each voxel was defined as the minimum of the neighborhood mean and midpoint between the neighborhood minimum and maximum. We repeated this parametric analysis in combination with commonly applied smoothing filters for HR-pQCT (Gaussian smoothing with σ = 0.8 and support = 1.0, or the Laplace–Hamming filter with recommended filter parameters^12^) to assess whether use of the Laplace–Hamming filter improved extraction of finer structural details. For each combination of parameters, we computed the Dice similarity coefficient (DSC)^20^ to evaluate spatial agreement with μCT, and the parameters that maximized DSC were selected as the optimal parameter set.

HR-pQCT images were then segmented using the standard, Laplace–Hamming, and AT segmentation methods. The standard segmentation method followed the standard guidelines for HR-pQCT image processing and analysis,^11^ using a Gaussian smoothing filter (σ = 0.8, support = 1.0) and global threshold of 320 mg HA/cm^3^. The Laplace–Hamming segmentation was performed using the recommended filter parameters (Laplace epsilon = 0.45, Hamming cutoff frequency = 0.3) and global threshold of 475 per mille.^12^ For each μCT and HR-pQCT scan, we computed mean trabecular thickness (Tb.Th), separation (Tb.Sp), and bone volume fraction (Tb.BV/TV) within the whole trabecular VOI. Trabecular number (Tb.N) was not computed as including all 4 trabecular parameters has been shown to reduce statistical power.^21^ Segmentation and quantification of Tb.Th, Tb.Sp, and Tb.BV/TV were performed using the ORMIR_XCT package.^22^ Measurement of Tb.Th, Tb.Sp, and Tb.BV/TV using the ORMIR_XCT package has been validated against IPL using an independent dataset (Supplemental Material S1).

### In vivo vs ex vivo segmentation comparison

To relate our ex vivo results to in vivo HR-pQCT scans, we used Bland–Altman plots to compare Tb.Th, Tb.Sp, and Tb.BV/TV between the standard and AT segmentation methods using *n* = 37 previously acquired in vivo HR-pQCT scans of healthy female trapezia^1,23^ (age: 48.5 ± 20.5 yr). HR-pQCT scanning was performed with an XtremeCT II scanner with 68 kV peak voltage, 1470 μA tube current, 100 ms integration time, and 60.7 μm isotropic voxel size.

### Statistical analysis

Groupwise distributions of bone microarchitecture parameters were tested for normality using the Shapiro–Wilk test and are reported as median and IQR due to non-normal distributions.

Spatial agreement between HR-pQCT and μCT segmentations was evaluated using DSC, mean Hausdorff distance and 95th percentile Hausdorff Distance (HD95),^24^ and average symmetric surface distance (ASSD).^25^ The DSC ranged from 0 to 1, with higher scores indicating better spatial overlap between HR-pQCT and μCT segmentations. Lower ASSD and Hausdorff distances indicate better agreement with μCT segmentations, with zero indicating perfect agreement. Segmentation quality metrics were computed using Python (v.3.8.5), SimpleITK^26^ (v2.0.2), and the seg-metrics^27^ package (v1.2.8).

Absolute and relative errors were computed for Tb.Th, Tb.Sp, and Tb.BV/TV between μCT and HR-pQCT segmentations. Due to non-independence of carpal bones from each specimen, we used a linear mixed-effects regression model and Bland–Altman analysis, adjusted for repeated measures,^28^ to assess the agreement in Tb.Th, Tb.Sp, and Tb.BV/TV. From regressions, we computed the repeated measures correlation coefficient^29^ (r) to quantify the strength of the association between HR-pQCT and μCT bone microarchitecture measures, while accounting for multiple bones from the same specimen. Pairwise differences in correlation coefficients between segmentation methods were evaluated using Steiger’s Z-test^30^ (α = .05). The proportional bias from Bland–Altman plots was evaluated by performing a linear regression of the bias and mean between HR-pQCT and μCT (α = .01). Biases from Bland–Altman plots were assessed for normality using the Shapiro–Wilk test and for sphericity using Mauchly’s test. Depending on these assumptions, either a repeated-measures ANOVA or Friedman test was used to compare error in bone microarchitecture parameters across segmentation methods. Post hoc pairwise comparisons between segmentation methods were performed using paired *t*-tests or Wilcoxon signed-rank tests (α = .05), consistent with ANOVA type. Pairwise tests were corrected for multiple comparisons using the Benjamini–Hochberg correction.

Statistical analyses were performed using Python (v.3.8.5), pingouin^31^ (v0.5.5) and statsmodels^32^ (v0.14.1). The seaborn^33^ (v0.11.2) and matplotlib^34^ (v3.7.2) packages were used for figures.

## Results

The 3 specimens used for AT parameter tuning were from donors with a mean age of 82.7 ± 4.6 yr, 2 of whom were female. The 5 hand specimens used for segmentation comparison were from donors with a mean age of 82.0 ± 6.4 yr, 3 of whom were female. The mean processing time for HR-pQCT segmentation was 0.11 ± 0.04 s for the standard method, 47.04 ± 21.63 s for the Laplace–Hamming method, and 47.70 ± 21.24 s for the AT method (16 core Apple M3 Max, 64 GB memory).

### Adaptive local threshold parameters

The optimal parameter set for carpal bones used a Laplace–Hamming filter, spherical kernel with radius of 6 voxels, lower threshold of 350 mg HA/cm^3^, and minimum structure size of 16 voxels. This resulted in a median DSC of 0.84 and IQR of 0.79-0.86 across carpal bones. Upon further inspection of the AT parameter optimization results, we found that segmentation performance was most sensitive to the choice of kernel radius, with larger values resulting in lower DSC. In contrast, the lower threshold parameter yielded high DSC across a broad range of values, with segmentation quality degrading with low values (eg, <200 mg HA/cm^3^). The minimum structure size had a minor influence on DSC and primarily affected the removal of small, isolated objects rather than overall segmentation performance.

### Spatial accuracy

**Figure 3:**
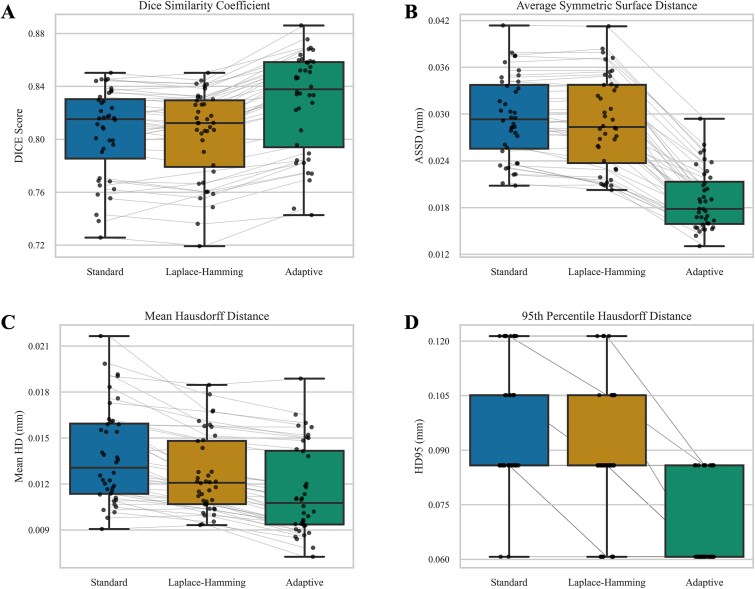
Segmentation quality metrics for the standard, Laplace–Hamming, and adaptive local threshold segmentation methods. Compared to the standard and Laplace–Hamming segmentations, the adaptive local threshold segmentations demonstrated increased Dice coefficients (A) and decreased average symmetric surface distances (ASSD) (B), mean Hausdorff distances (HD) (C), and 95th percentile Hausdorff distances (HD95) (D).

**Figure 4:**
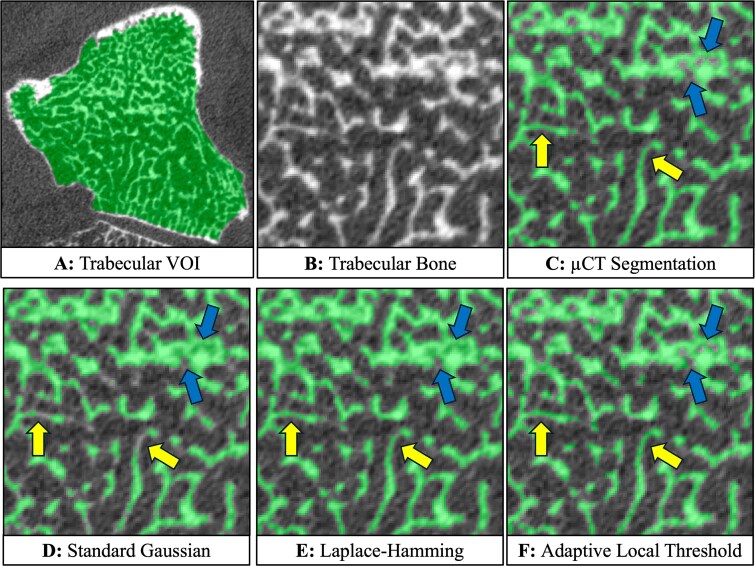
Example axial cross-section of a trapezium scanned with HR-pQCT showing the trabecular volume of interest (A) and trabecular bone segmentations for a magnified subregion of trabecular bone (B). The μCT segmentation was transformed to the HR-pQCT image and overlaid as the gold standard segmentation (C). The standard (D), Lapalce–Hamming (E), and adaptive local threshold (F) methods are shown for comparison. The adaptive local threshold segmentation improves detection of trabecular bone compared to the standard method (yellow arrows), without over-dilation that can be seen in the Laplace–Hamming method (blue arrows).

Overall, the AT method demonstrated higher spatial agreement with μCT segmentations across all metrics (Figure 3, Supplemental Material S2: Table S1). Notably, when using the AT method, the DSC, ASSD, and HD95 improved for all carpal bones analyzed, and mean Hausdorff distance improved for all but one carpal bone sample. On average, DSC increased by 3.1% with the AT method relative to both standard and Laplace–Hamming methods, indicating a marginally better volume segmentation. More notably, the metrics that capture accuracy of the segmented bone surface location resulted in strong improvements on average when using the AT method, compared to both the standard (decrease of 56.0% in ASSD, 20.1% in mean Hausdorff distance, and 41.3% in HD95) and Laplace–Hamming (decrease of 53.3% in ASSD, 9.6% in mean Hausdorff distance, and 28.8% in HD95) methods. Visual inspection of the segmentations showed that these metrics reflected an overall improvement of the AT method in capturing finer trabecular structure when compared to the standard method, without over-dilation that can occur with the Laplace–Hamming method (Figure 4).

### Bone microarchitecture accuracy

The AT method demonstrated the best overall agreement in Tb.Th, Tb.Sp, and Tb.BV/TV, relative to μCT (Table 1). All segmentation methods overestimated Tb.Th, however, the AT method yielded the lowest absolute (0.05 mm, IQR: 0.04-0.06 mm) and relative (19.6%) errors, more than halving the error observed in the standard (absolute error: 0.14 mm, IQR: 0.11-0.17 mm, relative error: 56.2%) and Laplace–Hamming (absolute error: 0.13 mm, IQR: 0.10-0.16 mm, relative error: 52.2%) methods. Similarly, the AT method produced the lowest errors for Tb.Sp and Tb.BV/TV (relative errors of −1.8% and 8.5%, respectively), compared to the standard (relative errors of: 13.1% and 23.8%, respectively) and Laplace–Hamming (relative errors of 2.7% and 33.8%, respectively) methods.

**Table 1:** Trabecular thickness (Tb.Th), trabecular separation (Tb.Sp), and trabecular bone volume fraction (Tb.BV/TV) values (median [IQR]) from the ground-truth μCT segmentation and standard, Laplace–Hamming, and adaptive local threshold segmentations, averaged across all carpal bones (*n* = 64). Errors are provided as median absolute error [IQR] (percentage error).

	μCT	HR-pQCT standard		HR-pQCT Laplace–Hamming		HR-pQCT adaptive threshold	
	Median [IQR]	Median [IQR]	Error [IQR] (%)	Median [IQR]	Error [IQR] (%)	Median [IQR]	Error [IQR] (%)
**Tb.Th (mm)**	0.25 [0.23-0.28]	0.39 [0.33-0.43]	0.14 [0.11-0.17] (56.2%)	0.38 [0.33-0.41]	0.13 [0.10-0.16] (52.2%)	0.30 [0.28-0.31]	0.05 [0.04-0.06] (19.6%)
**Tb.Sp (mm)**	0.56 [0.53-0.59]	0.66 [0.62-0.69]	0.08 [0.06-0.11] (13.1%)	0.59 [0.57-0.64]	0.02 [0.01-0.03] (2.7%)	0.57 [0.55-0.61]	0.02 [0.01-0.02] (−1.8%)
**Tb.BV/TV**	0.34 [0.32-0.37]	0.41 [0.36-0.45]	0.08 [0.04-0.11] (23.8%)	0.45 [0.40-0.48]	0.12 [0.09-0.14] (33.8%)	0.37 [0.34-0.38]	0.03 [0.02-0.04] (8.5%)

**Figure 5:**
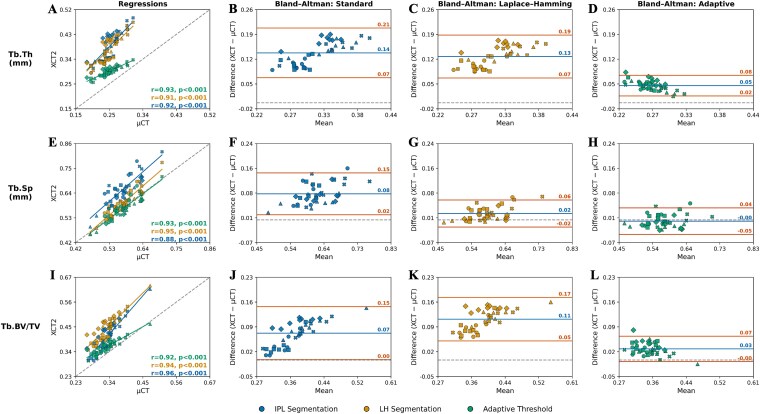
Regression (A, E, and I) and Bland–Altman (B-D, F-H, and J-L) plots for trabecular thickness (Tb.Th), trabecular separation (Tb.Sp), and trabecular bone volume fraction (Tb.BV/TV) between μCT and each HR-pQCT segmentation method. For regression plots, the line of unity is represented with the dashed line, and the repeated measures correlation coefficient (r) is provided for each HR-pQCT segmentation method. For Bland–Altman plots, the 95% limits of agreement are represented with solid orange lines, and the mean bias is represented by a solid blue line. Bland–Altman plots have been adjusted for repeated measures. Results from each specimen are plotted with different markers.

For all trabecular parameters, strong positive correlations were observed between μCT and all HR-pQCT segmentation methods (r > 0.88, *p* < .001, Figure 5A, E, and I). However, across all methods, the slopes from the linear mixed-effects regressions were significantly different from one (*p* < .001), while the intercepts were inconsistent in terms of the parameters and segmentation method that resulted in significant differences from zero (Table 2). Bland–Altman analysis showed that the AT method had the lowest mean bias and narrowest limits of agreement across all trabecular properties compared to the Laplace–Hamming and standard methods (Figure 5). Notably, for Tb.BV/TV and Tb.Sp the AT method resulted in near-zero proportional biases, whereas the standard and Laplace–Hamming methods both overestimated these metrics. The AT method significantly reduced the bias in Tb.Th (*p* < .01), Tb.Sp (*p* < .01), and Tb.BV/TV (*p* = .03) compared to the standard method, and in Tb.Th (*p* < .01) and Tb.BV/TV (*p* < .01) compared to the Laplace–Hamming method. Although a proportional bias for Tb.Th and Tb.BV/TV was still observed for the AT method, it was reduced by more than half compared to the other 2 segmentation methods (Tb.Th bias of 0.05 mm vs 0.13-0.14 mm, Tb.BV/TV bias of 0.03 vs 0.07-0.11). However, a significant proportional bias was consistently found for Tb.Th and Tb.BV/TV with all segmentation methods (*p* < .05), and for Tb.Sp in the standard and Laplace–Hamming methods (*p* < .001), indicating errors were not constant with increasing measurement values regardless of the segmentation method used.

**Table 2:** Slope, intercepts, and repeated-measures correlation coefficients (r) from the linear mixed-effects regressions between bone microarchitecture measures from μCT and HR-pQCT.

	Slope	Intercept	r
**Standard**			
**Tb.Th**	**1.43**	0.03	**0.92**
**Tb.Sp**	**1.19**	−0.03	**0.89**
**Tb.BV/TV**	**1.55**	**−0.11**	**0.96**
**Laplace**–**Hamming**			
**Tb.Th**	**1.31**	0.05	**0.91** ^*^
**Tb.Sp**	**1.18**	**−0.08**	**0.95** ^*^
**Tb.BV/TV**	**1.32**	0.005	**0.94** ^*^
**Adaptive local threshold**			
**Tb.Th**	**0.62**	**0.14**	**0.93** ^**†**^
**Tb.Sp**	**1.01**	−0.01	**0.93** ^*,**†**^
**Tb.BV/TV**	**0.75**	**0.12**	**0.90** ^*,**†**^

Slopes significantly different from 1, intercepts significantly different from 0, and significant r values are bolded. Post-hoc analyses between HR-pQCT segmentation methods were performed using Steiger’s Z test to assess if correlation strength significantly differed between methods. ^*^p < .001 compared to the standard method, and ^†^*p* < .001 compared to the Laplace–Hamming method. Abbreviations: Tb.Th: trabecular thickness, Tb.Sp: trabecular separation, and Tb.BV/TV: trabecular bone volume fraction.

### In vivo vs ex vivo segmentation comparison

**Figure 6:**
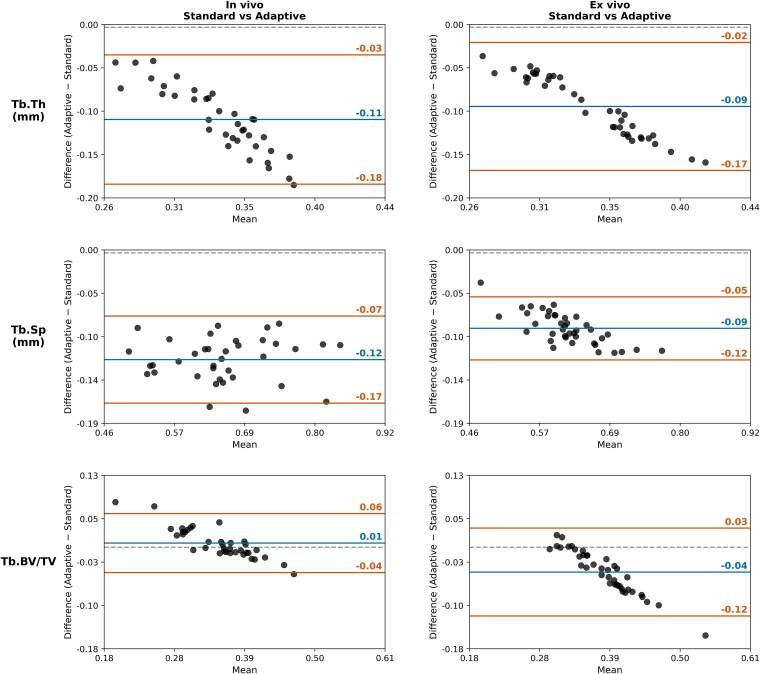
Bland–Altman plots for trabecular thickness (Tb.Th), trabecular separation (Tb.Sp), and trabecular bone volume fraction (Tb.BV/TV) between the standard and adaptive local threshold segmentation methods applied to in vivo HR-pQCT scans of trapezia (*n* = 37, left) and ex vivo HR pQCT scans of carpal bones (*n* = 40) from five human cadaveric hands (right). Similar patterns were observed between in vivo and ex vivo datasets, suggesting that the adaptive local threshold method reduces systematic over- or under-estimation of trabecular bone microarchitecture in vivo, relative to the current standard method. The 95% limits of agreement are represented with solid orange lines, and the mean bias is represented by a solid blue line.

In both datasets, the standard method produced higher estimates of Tb.Th and Tb.Sp than the AT method, resulting in lower biases (Figure 6). For Tb.BV/TV, the standard method yielded higher values in the cadaveric dataset (bias = −0.04), whereas the AT method yielded higher Tb.BV/TV estimates with the in vivo data (bias = 0.01).

## Discussion

In this study, we compared the accuracy of an AT segmentation method with the standard and Laplace–Hamming segmentation methods in second-generation HR-pQCT scans of carpal bones. The AT method demonstrated the best overall agreement with μCT, providing improved spatial agreement and greater accuracy in trabecular bone microarchitecture compared. Further, when comparing trabecular bone microarchitecture between the standard and AT methods using in vivo HR-pQCT scans, we observed similar trends to those seen in the cadaveric scans. These findings suggest that AT improves segmentation of trabecular bone in carpal bones that consist of finer structural details.

Notably, all metrics evaluating accuracy of segmentation relative to μCT were improved with the AT method. Measures that captured accuracy of the bone surface resulted in the highest improvements overall, emphasizing how subtle, but important details for advanced analytical methods are better captured with an AT. However, it is noted that only a marginal increase in DSC was observed, indicating that error in the segmentation volume persists. This may be attributed to the interpolation artifacts that are introduced when transforming μCT segmentations to the downscaled HR-pQCT image space. This is an inherent resolution-based limitation, and likely accounts for a large portion of the remaining inaccuracy between HR-pQCT and μCT methods. Further, although all registrations were visually inspected for accuracy, slight misalignments between μCT and HR-pQCT images may persist which could impact spatial agreement of all segmentation methods. Regardless, it is worth noting that segmentation accuracy improved for all analyzed bone samples when using the AT method, although the magnitude of improvement varied across samples. This is expected as the standard segmentation method is tuned to perform well for most bone, and thus in many cases is suitable for capturing the trabecular structure. However, for irregular bone structures, these global thresholds tend to misrepresent finer structural details and thus, we see greater improvements with the AT method.

Our findings in the carpals agree with previous HR-pQCT studies demonstrating systematic overestimation of Tb.Th and Tb.BV/TV in the distal radius and tibia. Sadoughi et al. compared the accuracy of the standard and Laplace–Hamming segmentation methods in the distal radius and tibia and found that both methods overestimate Tb.Th and Tb.BV/TV, while underestimating Tb.Sp.^12^ Similarly, Zhou et al. reported that the Laplace–Hamming method improved the accuracy of both bone microarchitecture measures and estimates of bone strength from micro-finite element analysis (μFEA) in the distal radius and tibia, also noting an underestimation of Tb.Sp.^35^ Mys et al. compared the accuracy of the standard and AT methods in the distal radius, again showing an overestimation of Tb.Th and Tb.BV/TV, and underestimation of Tb.Sp with both methods.^14^ In line with these findings, Quintiens et al. reported that AT in photon-counting CT scans of carpal bones showed improved accuracy with μCT compared to global thresholding.^15^ Our results show that the AT method significantly reduces the overestimation of Tb.Th and Tb.Sp compared to the standard method and significantly reduces the overestimation of Tb.Th compared to the Laplace–Hamming method, highlighting its improved accuracy. Methodologically, our AT algorithm differed from those previously described,^14,15^ as we applied a Laplace–Hamming filter and used a spherical kernel with radius of 6 voxels, lower threshold of 350 mg HA/cm^3^, and classified voxels as bone using the minimum of the local mean and the midpoint between the neighborhood minimum and maximum. Further, our AT algorithm is fully open-source as part of the ORMIR_XCT package.^22^

Visual inspection of the HR-pQCT segmentations showed that the AT method better preserved narrow spaces between adjacent trabeculae that were frequently merged by the standard and Laplace–Hamming methods, resulting in lower mean measures of trabecular properties and improved agreement with μCT. We restricted our analysis to trabecular microarchitecture as, unlike long bones, the carpal bones have a thin, sometimes discontinuous, cortical shell, resulting in difficulties applying existing HR-pQCT workflows for separating cortical and trabecular regions.^9,10^ Nevertheless, the AT method’s improved detection of fine trabeculae and ability to preserve small spaces between trabeculae suggests potential to improve accuracy of cortical bone microarchitecture measures from HR-pQCT scans. However, the AT method used in this study was optimized for trabecular bone and may not be directly applicable to cortical bone.

The reliance of the standard and Laplace–Hamming methods on fixed global thresholds represents an inherent sensitivity to threshold selection. Modification of the fixed thresholds used in the standard and Laplace–Hamming methods may improve their segmentation accuracy relative to μCT in carpal bones; however, it may be challenging to balance the detection of fine trabeculae with overdilation. In contrast, the AT method determines local thresholds, allowing for greater robustness to anatomical complexity and spatial variations in bone density. To investigate the sensitivity of fixed global threshold values on the accuracy of the standard and Laplace–Hamming methods, we performed an exploratory sensitivity analysis on *n* = 15 carpal bones. Global thresholds for the standard method were varied from 280 to 360 mg HA/cm^3^, in increments of 10 mg HA/cm^3^, and from 435 to 515 per mille for the Laplace–Hamming method, in increments of 10 per mille. For each threshold value, we computed the same segmentation quality metrics and trabecular microarchitecture properties described previously. Across the tested thresholds, the AT method consistently yielded the smallest errors in Tb.Th and Tb.Sp relative to μCT. For Tb.BV/TV, the standard method produced slightly lower errors at threshold values of 340, 350, and 360 mg HA/cm^3^. However, these reductions in Tb.BV/TV error were accompanied by degraded spatial overlap metrics (Supplementary Material S2: Figure 1), including lower DSC and higher HD95 and ASSD. These findings indicate that although adjustment of global thresholds may improve accuracy of select trabecular microarchitecture parameters, these improvements come at the expense of spatial accuracy. Taken together, this analysis suggests that the AT method provides greater robustness to anatomical complexity and spatial variations in bone density in carpal bones compared to the standard and Laplace–Hamming methods.

As an additional analysis, we examined whether the differences observed between the standard and AT methods were also present in vivo*.* Across all trabecular parameters, we saw similar patterns between the in vivo and ex vivo Bland-Altman plots, with the standard segmentation method producing higher estimates of Tb.Th and Tb.Sp. The agreement in biases across both datasets suggests that the method-dependent differences are systematic rather than specimen-specific, supporting the use of the AT method for in vivo HR-pQCT scans. However, our in vivo data was limited to a single carpal bone, which may not capture the variability represented across the different cadaveric carpal bones.

This study was not without its limitations. First, the parameter optimization was performed on three specimens, and further validation in a larger sample, and across a wider age range would be preferred to ensure broader generalizability of these parameters. However, access to cadaveric samples is challenging and the presented dataset is reasonably comparable to other cadaveric validation studies. To minimize the effect of having a limited dataset, we selected specimens for parameter optimization to best capture the variability available, ensuring a balanced representation of male and female samples, and representation of youngest and oldest available samples. Second, we restricted our analysis to trabecular bone as separating cortical and trabecular bone in carpal bones remains challenging. Although the AT method improved trabecular segmentation, its performance for cortical bone segmentation has not been established. The thin and non-uniform cortical shell in carpal bones is challenging for automated segmentation methods, and more work is needed to develop robust methods to delineate cortical and trabecular bone in carpal bones. Finally, while the AT method reduced errors in trabecular microarchitecture relative to μCT, complete agreement with μCT was not possible. An error of 19.6% in Tb.Th was found with the AT method, however, this reflects a fundamental limitation of HR-pQCT spatial resolution, as some trabeculae with sub-resolution thickness cannot be fully resolved. Bland-Altman analysis demonstrated a significant proportional bias for Tb.Th and Tb.BV/TV across all segmentation methods, indicating that error varied with mean trabecular thickness and density. For the AT method, Tb.Th bias decreased with increasing mean Tb.Th. In contrast, Tb.Th bias for the standard and Laplace–Hamming methods increased with increasing mean Tb.Th. This suggests that the standard and Laplace–Hamming methods tend to overestimate Tb.Th in structurally denser bone. Moreover, absolute and relative Tb.Th errors across individual carpal bones were consistently lower with the AT method (Supplementary Material S2: Table S2), suggesting that the AT method is more robust across carpal bones with differing morphologies and trabecular densities.

In conclusion, the AT segmentation method improved accuracy of detailed trabecular bone structure extracted from HR-pQCT scans of carpal bones compared to the standard and Laplace–Hamming segmentation methods. The AT method presented allows for improved detection of thin, low-density trabeculae that are often lost with global approaches. Although a local adaptive method requires additional computational time and added segmentation complexity, this is expected to benefit a broad range of applications that seek to employ more advanced analytical techniques that require accurate extraction of the bone surface.

## Supplementary Material

ziag054_Supplemental_Files

## Data Availability

Image data used in this study are openly available at: https://doi.org/10.48804/DWF4RG. Software used for image segmentation and computation of bone microarchitecture are openly available at: https://github.com/ORMIR-XCT/ORMIR-XCT. Software used for image registration is openly available at: https://github.com/PaediatricMSKImaging/XCT2-Carpal-Stack-Registration.
