# Supplementary material for: Improved segmentation accuracy in high-resolution peripheral quantitative computed tomography scans of carpal bones using adaptive local thresholding
Source: JBMR Plus. 2026 Mar 30;10(6):ziag054. doi: 10.1093/jbmrpl/ziag054 (PMC13160417; doi:10.1093/jbmrpl/ziag054)
Supplement: ziag054_Supplemental_Files [file ziag054_supplemental_files.zip › Supplementary_Material_1_Rev1_clean_ziag054.docx]

**Supplementary Material 1:** Validation of ORMIR_XCT Trabecular Bone Microarchitecture Measures Against IPL

# Materials and Methods

## Cohort

The study population included 114 young, healthy individuals aged 22 to 37 years (54 females, 60 males) who were recruited through local advertisement at the University of Bern and the University Hospital Bern to create a normative reference dataset representative of the Swiss population. More details on the study demographics can be found in Table 1. The study was conducted under the approval of the ethics committee of the Canton Bern (BASEC ID: 2017-00882), all participants provided written informed consent. More details are presented by Stuck et al [1].

**Supplementary Table 1:** Age (years), BMI (kgm−3), and Femoral neck aBMD (gcm−2).

|  | Females (*n* =54) | Males (*n* =60) | p-value* |
| --- | --- | --- | --- |
| Age (years) |  |  |  |
| Mean (sd) | 25.17 (4.72) | 25.98 (4.37) | 0.34 |
| Median [lq, uq] | 24.00 [21.00, 28.00] | 25.00 [22.00, 28.25] | 0.18 |
| BMI (kg/m²) |  |  |  |
| Mean (sd) | 22.40 (2.76) | 23.91 (3.03) | 0.006 |
| Median [lq, uq] | 22.22 [20.95, 23.11] | 23.38 [22.13, 25.88] | 0.004 |
| Femoral neck aBMD (g/cm²) |  |  |  |
| Mean (sd) | 0.85 (0.12) | 0.94 (0.17) | p<0.001 |
| Median [lq, uq] | 0.83 [0.78, 0.94] | 0.95 [0.81, 1.06] | 0.002 |

*Student’s t-test for continuous variables presented with mean (sd) and the WilcoxonMann-Whitney test for continuous variables presented with median [lq, uq]. *sd, standard deviation; lq, lower quartile; uq, upper quartile; BMI, body mass index; aBMD, areal bone mineral density*.

## Image acquisition

A second-generation HR-pQCT scanner (XtremeCT II, Scanco Medical AG, Brüttisellen, Switzerland) was used to scan the non-dominant side of both radius and tibia (or, in case of fractures, on the non-fractured side). The imaging protocol was consistent with the standard clinical workflow, except for the size and positioning of the region of interest (ROI). Images were acquired at a voxel size of 60*.*7 µm using standard imaging settings (*U_p_* = 68 kV, I = 1480 µA, integration time t = 43ms). A custom multi-stack protocol was used to increase the region of interest along the distal metaphysis from 10*.*1 mm to 20*.*2 mm (336 slices) and 30*.*3 mm (504 slices) at the radius and tibia, respectively. In order to compare the newly proposed microarchitectural evaluation, the multi-stack sections were subsequently cropped to their corresponding single-stack sections.

## Image processing and analysis

The images were first cropped to their corresponding single-stack equivalent using the relative offset method [2] to retrieve the positioning of the ROI. When not fully covered, the most distal stack was kept. To estimate the ground truth, image processing was performed using the scanner standard workflow (IPL Scanco Module 64-bit, V5.16). Bone microarchitectural parameters were estimated using the open-source Python implementation proposed by Kuczynski et al. [3]. The parameters of interest were trabecular bone volume fraction (Tb.BV/TV), trabecular thickness (Tb.Th), and trabecular separation (Tb.Sp). Briefly, Tb.BV/TV was calculated as the ratio of segmented bone volume over the total mask volume. Trabecular thickness and separation were computed using the algorithm proposed by Hildebrand and Ruegsegger (1997) [4], which estimates local thickness based on the maximum inscribed sphere diameter at each voxel within the segmented bone structure (Tb.Th) or the segmented marrow space (Tb.Sp).

In order to compare the implementation and assess the impact of two different algorithmic optimizations, four configuration combinations of the thickness estimation algorithm were evaluated. The two independent features are (1) skeletonization, which restricts thickness calculation to the medial axis of the microstructure to reduce computational requirements, and (2) oversampling of the distance transform to align the output of common implementations with the theoretical definition proposed by Hildebrand and Ruegsegger. The four configurations were systematically tested against the ground truth by means of linear regression and Bland-Altman plots. The aim was to determine which configuration corresponds most accurately to the standard output provided by the scanner manufacturer, and to determine which configuration produces the most accurate distance transform image by means of visual inspection.

# Results

The comparison between the open-source Python implementation proposed by Kuczynski et al. and the scanner’s standard patient evaluation revealed excellent agreement for Tb.BVTV ($R^{2}=1.00$), Tb.Th ($R^{2}=0.99$), and Tb.Sp ($R^{2}=1.00$) for all four parameters combinations. The nearest prediction with the gold standard of IPL was achieved with both oversampling and skeletonization deactivated (see Table 2). The microarchitectural parameters Tb.Th showed a systematic overestimation by the open-source method, as indicated by the slope of 1.23 in the linear regression (See Figure 1). Findings were confirmed by Bland-Altman plots, demonstrating negligible bias for Tb.BV/TV, but a small bias in Tb.Th (mean difference = -0.04 mm) and in Tb.Sp (mean difference = -0.08 mm) (see Figure 2). The limits of agreement were the narrowest for Tb.BV/TV, and widest for Tb.Sp. No systematic differences were observed between measurement sites across any of the evaluated parameters. The visual inspection yielded consistent results (see Figure 3).


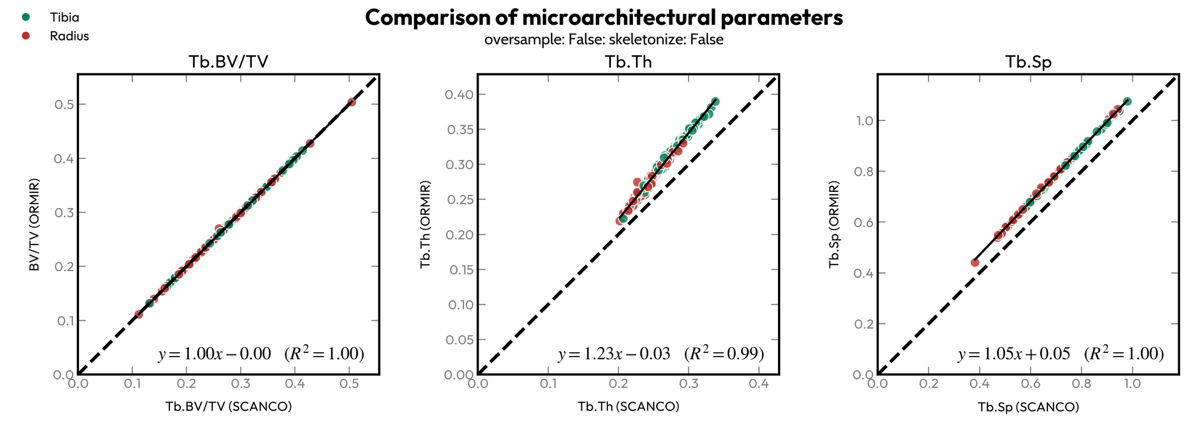


Figure 1: Comparison between the ground truth and the ORMIR implementation.


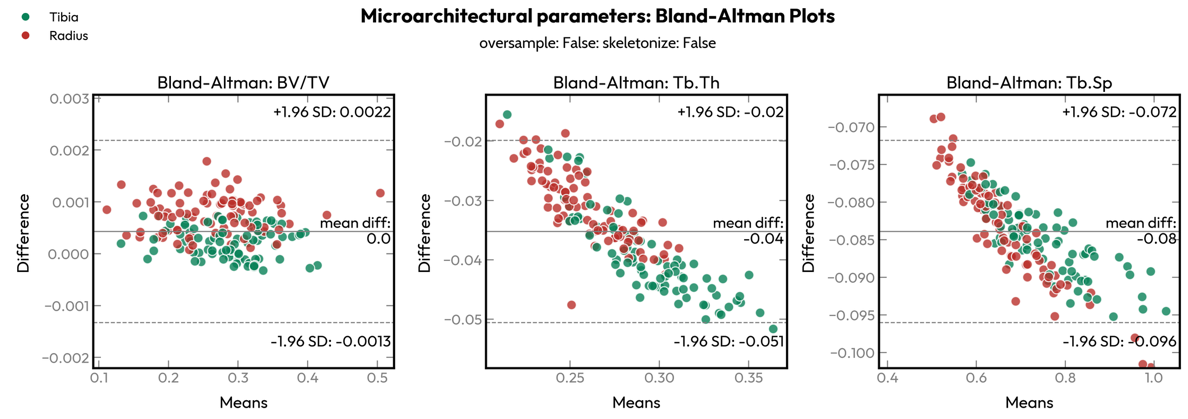


**Supplementary Figure 2:** Bland-Altman plots between the ground truth and the ORMIR implementation.

**Supplementary Table 2:** Results matrix comparing the four combinations.

| Oversample | Skeletonize | Tb.Th | | | Tb.Sp | | |
| --- | --- | --- | --- | --- | --- | --- | --- |
|  |  | *a* | *b* | *R^2^* | *a* | *b* | *R^2^* |
| False | False | 1.23 | -0.03 | 0.99 | 1.05 | 0.05 | 1.00 |
| False | True | 1.15 | 0.00 | 0.99 | 1.02 | 0.07 | 1.00 |
| True | False | 1.38 | -0.09 | 0.99 | 1.17 | 0.00 | 1.00 |
| True | True | 1.36 | -0.09 | 0.99 | 1.14 | 0.01 | 1.00 |

Results are presented in the form *y* = *a* ⋅ *x* + *b* (*R*^2^_)_. Tb.Th: trabecular thickness; Tb.Sp: trabecular separation.

|  |  | **Skeletonization** | |
| --- | --- | --- | --- |
| **Oversampling** |  | **False** | **True** |
|  | **False** | **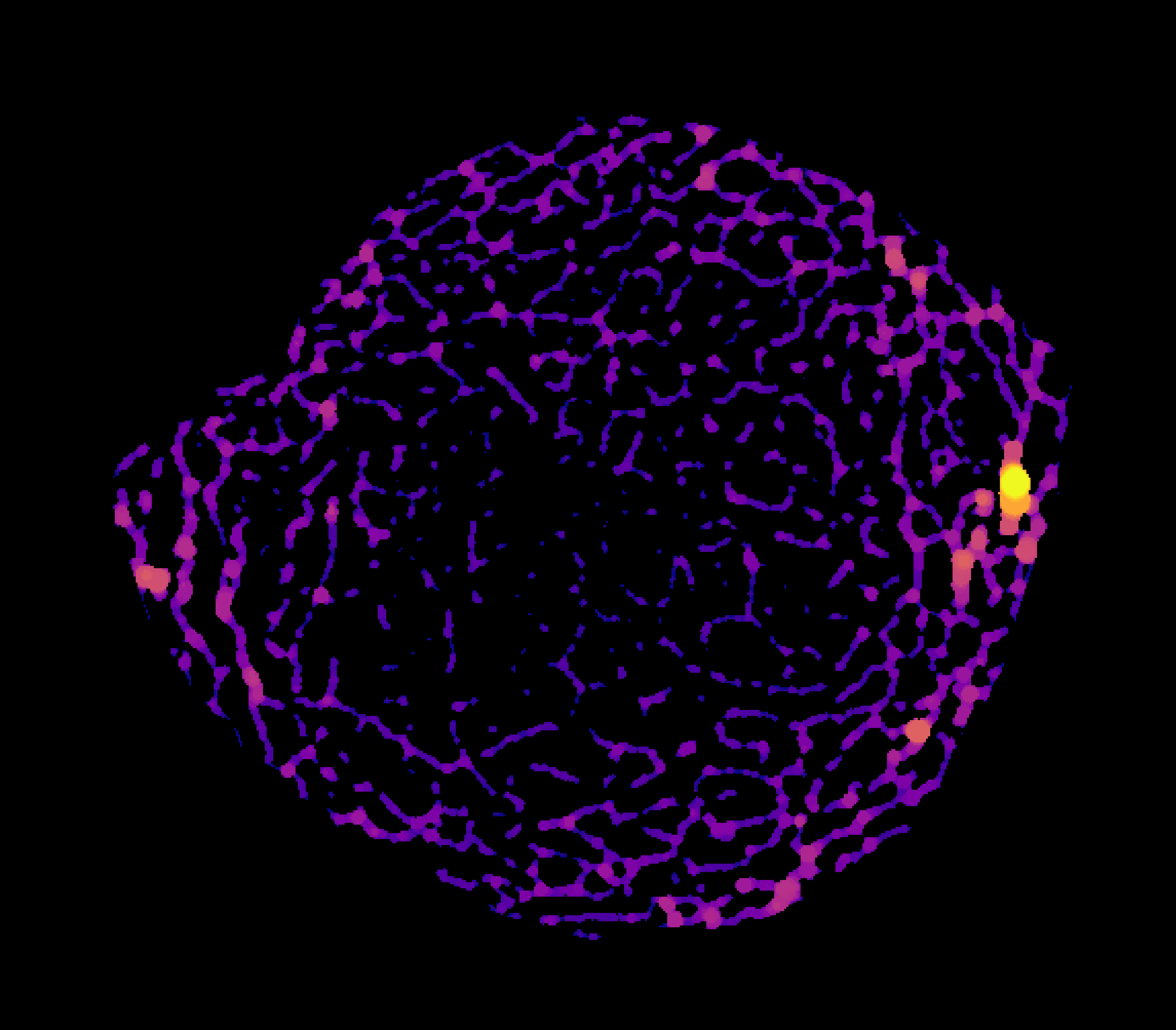** | **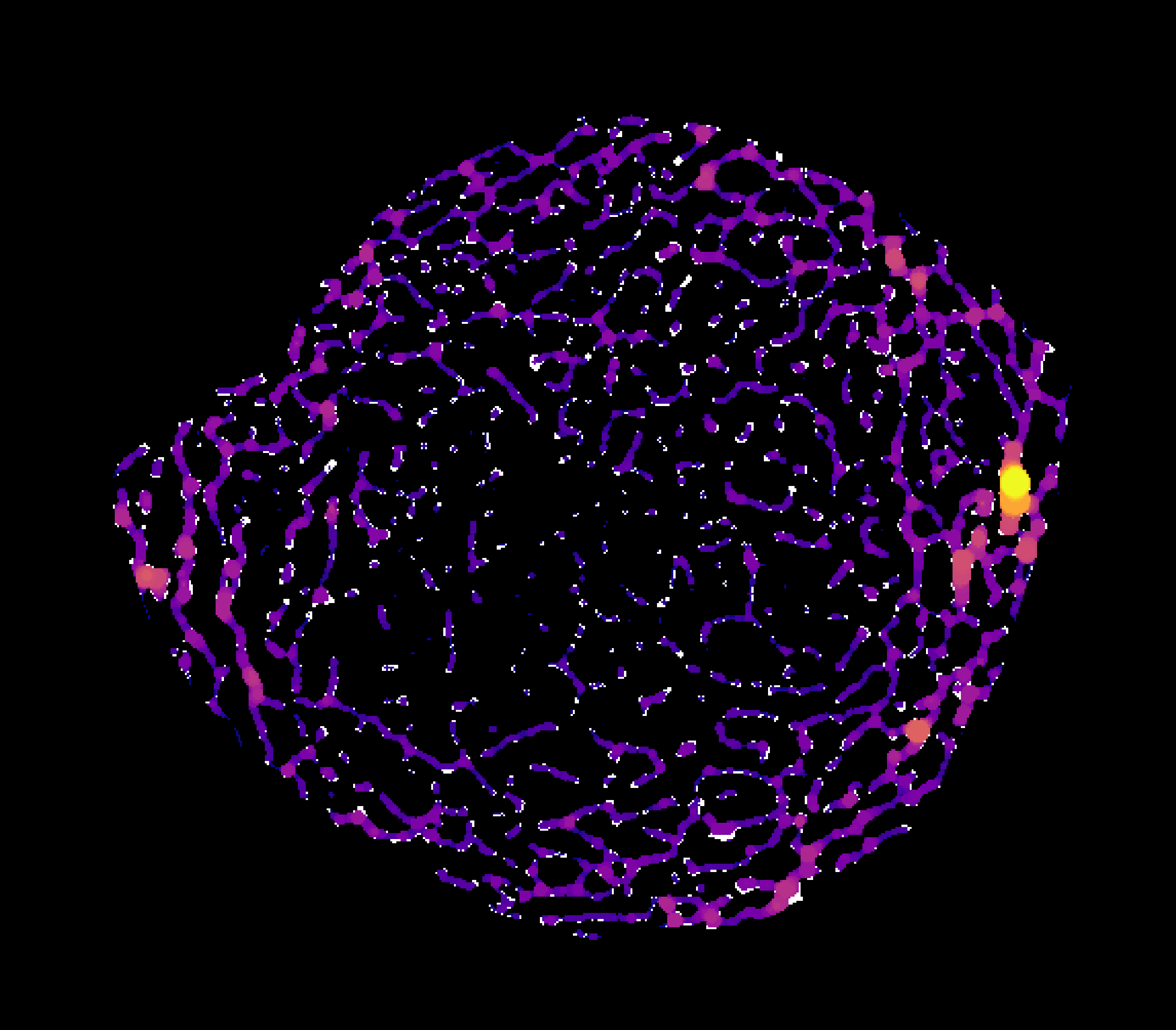** |
|  | **True** | **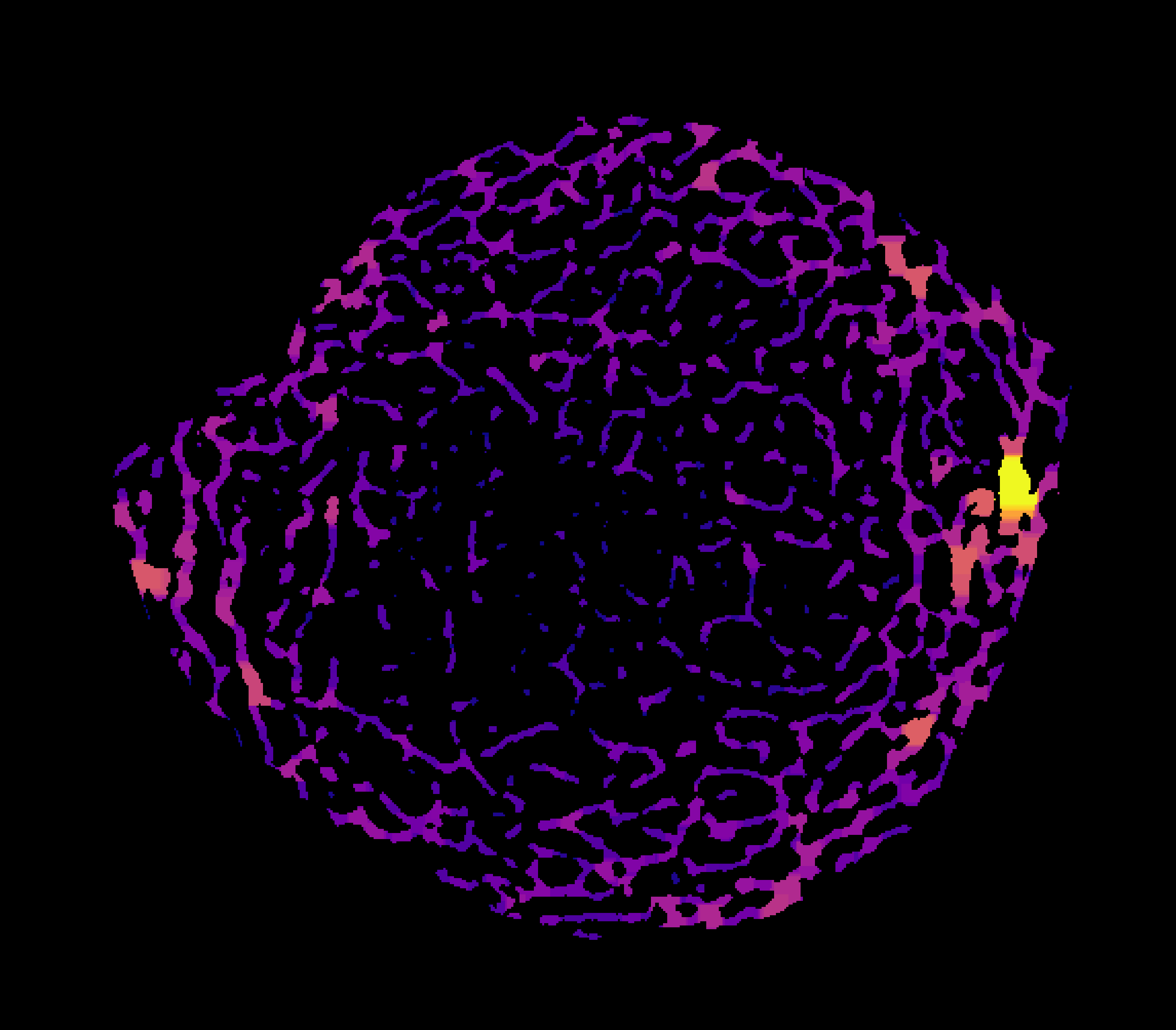** | **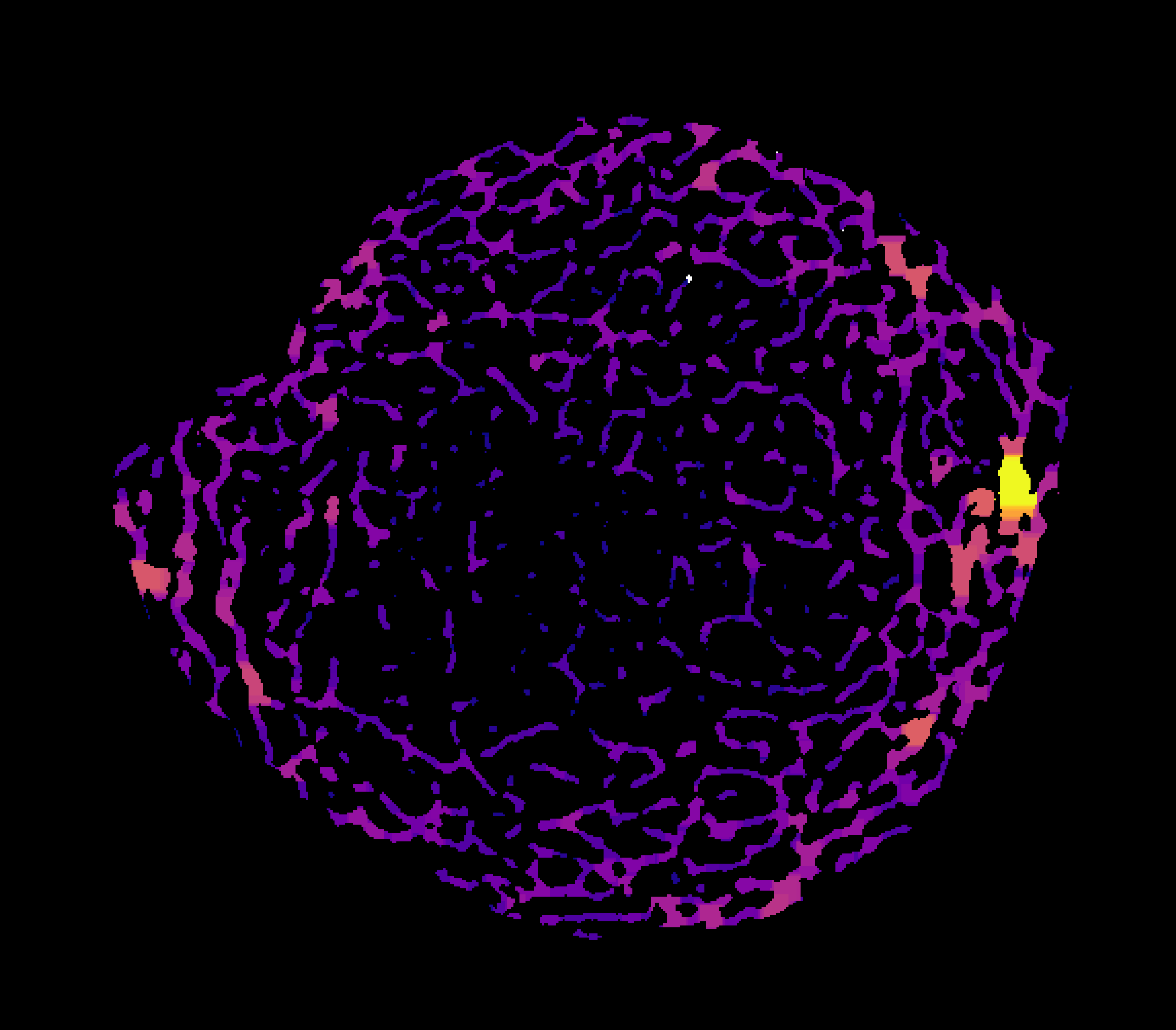** |

**Supplementary Figure 3:** Visual inspection of the microarchitectural evaluation

# **References**

[1] A. K. Stuck, D. Schenk, P. Zysset, L. Bütikofer, A. Mathis, and K. Lippuner, ‘Reference values and clinical predictors of bone strength for HR-pQCT-based distal radius and tibia strength assessments in women and men’, *Osteoporosis International*, vol. 31, no. 10, pp. 1913–1923, Oct. 2020, doi: 10.1007/s00198-020-05405-0.

[2] S. Bonaretti, S. Majumdar, T. F. Lang, S. Khosla, and A. J. Burghardt, ‘The comparability of HR-pQCT bone measurements is improved by scanning anatomically standardized regions’, *Osteoporosis International*, vol. 28, no. 7, pp. 2115–2128, July 2017, doi: 10.1007/s00198-017-4010-7.

[3] M. T. Kuczynski *et al.*, ‘ORMIR_XCT: A Python package for high resolutionperipheral quantitative computed tomography image processing’, *JOSS*, vol. 9, no. 97, p. 6084, May 2024, doi: 10.21105/joss.06084.

[4] T. Hildebrand and P. Rüegsegger, ‘A new method for the model‐independent assessment of thickness in three‐dimensional images’, *Journal of Microscopy*, vol. 185, no. 1, pp. 67–75, Jan. 1997, doi: 10.1046/j.1365-2818.1997.1340694.x.
