# Supplementary material for: Improved segmentation accuracy in high-resolution peripheral quantitative computed tomography scans of carpal bones using adaptive local thresholding
Source: JBMR Plus. 2026 Mar 30;10(6):ziag054. doi: 10.1093/jbmrpl/ziag054 (PMC13160417; doi:10.1093/jbmrpl/ziag054)
Supplement: ziag054_Supplemental_Files [file ziag054_supplemental_files.zip › Supplementary_Material_2_Rev1_clean_ziag054.docx]

Supplementary Material 2

***Supplementary Table 1:*** *Segmentation quality metrics for the standard, Laplace-Hamming, and adaptive local threshold segmentation methods (median [interquartile range]). Results are averaged across all bones and specimens (n=5 hand specimens, resulting in n=40 carpal bones).*

|  | **Standard Gaussian Segmentation** | **Laplace-Hamming Segmentation** | **Adaptive Threshold Segmentation** |
| --- | --- | --- | --- |
| DSC [0-1] | 0.82 [0.79-0.83] | 0.81 [0.78-0.83] | 0.84 [0.79-0.86] |
| Mean HD (mm) | 0.013 [0.011-0.016] | 0.012 [0.011-0.015] | 0.011 [0.009-0.014] |
| 95^th^ Percentile HD (mm) | 0.086 [0.086-0.105] | 0.086 [0.086-0.105] | 0.061 [0.061-0.086] |
| ASSD (mm) | 0.029 [0.026-0.34] | 0.028 [0.024-0.034] | 0.018 [0.016-0.021] |

***Supplemental Table 2:*** *Trabecular thickness (Tb.Th), trabecular separation (Tb.Sp), and trabecular bone volume fraction (Tb.BV/TV) values (median [interquartile range]) from the standard, Laplace-Hamming, and adaptive local threshold segmentations for each bone and all bones across all specimens. Errors are provided as median absolute error [interquartile range] (median relative error*)*. IQR: interquartile range.*

|  | **Tb.Th (mm)** | | **Tb.Sp (mm)** | | **Tb.BV/TV** | |
| --- | --- | --- | --- | --- | --- | --- |
|  | **Median [IQR]** | **Absolute Error [IQR] (%)** | **Median [IQR]** | **Absolute Error [IQR] (%)** | **Median [IQR]** | **Absolute Error [IQR] (%)** |
| *Standard* |  |  |  |  |  |  |
| Capitate | 0.44 [0.35-0.47] | 0.17 [0.10-0.18] (57.2%) | 0.63 [0.62-0.63] | 0.07 [0.06-0.08] (13.4%) | 0.49 [0.37-0.50] | 0.11 [0.04-0.11] (26.9%) |
| Hamate | 0.42 [0.33-0.43] | 0.15 [0.10-0.15] (51.6%) | 0.67 [0.67-0.73] | 0.08 [0.08-0.13] (14.4%) | 0.44 [0.32-0.44] | 0.08 [0.02-0.10] (20.8%) |
| Lunate | 0.40 [0.38-0.41] | 0.15 [0.14-0.17] (57.1%) | 0.61 [0.61-0.67] | 0.05 [0.04-0.06] (8.6%) | 0.42 [0.42-0.43] | 0.09 [0.09-0.10] (26.3%) |
| Pisiform | 0.32 [0.31-0.33] | 0.12 [0.10-0.13] (60.6%) | 0.64 [0.60-0.65] | 0.12 [0.07-0.12] (22.7%) | 0.31 [0.30-0.35] | 0.03 [0.03-0.08] (11.6%) |
| Scaphoid | 0.42 [0.37-0.43] | 0.16 [0.13-0.17] (60.3%) | 0.62 [0.59-0.71] | 0.05 [0.04-0.08] (8.9%) | 0.45 [0.44-0.46] | 0.10 [0.10-0.11] (29.0%) |
| Trapezium | 0.39 [0.33-0.41] | 0.14 [0.10-0.16] (54.0%) | 0.71 [0.68-0.72] | 0.10 [0.08-0.11] (17.3%) | 0.36 [0.35-0.42] | 0.06 [0.03-0.07] (17.8%) |
| Trapezoid | 0.41 [0.34-0.43] | 0.15 [0.10-0.16] (54.2%) | 0.65 [0.64-0.68] | 0.06 [0.06-0.07] (10.3%) | 0.40 [0.38-0.45] | 0.06 [0.06-0.09] (20.3%) |
| Triquetrum | 0.41 [0.33-0.42] | 0.15 [0.11-0.18] (55.9%) | 0.66 [0.65-0.66] | 0.11 [0.08-0.11] (20.0%) | 0.37 [0.33-0.42] | 0.08 [0.04-0.09] (22.9%) |
| All | 0.39 [0.33-0.43] | 0.14 [0.11-0.17] (56.2%) | 0.66 [0.62-0.69] | 0.08 [0.06-0.11] (13.1%) | 0.41 [0.36-0.45] | 0.08 [0.04-0.11] (23.8%) |
| *Laplace-Hamming* |  |  |  |  |  |  |
| Capitate | 0.43 [0.35-0.45] | 0.16 [0.09-0.16] (52.9%) | 0.57 [0.57-0.58] | 0.02 [0.02-0.02] (3.0%) | 0.51 [0.41-0.52] | 0.13 [0.08-0.14] (33.6%) |
| Hamate | 0.40 [0.33-0.41] | 0.13 [0.10-0.14] (44.9%) | 0.61 [0.60-0.65] | 0.02 [0.02-0.05] (3.8%) | 0.47 [0.36-0.47] | 0.10 [0.07-0.13] (27.8%) |
| Lunate | 0.39 [0.37-0.40] | 0.14 [0.13-0.16] (54.0%) | 0.57 [0.54-0.62] | 0.00 [0.00-0.00] (-0.2%) | 0.46 [0.46-0.46] | 0.12 [0.12-0.14] (35.6%) |
| Pisiform | 0.31 [0.30-0.33] | 0.12 [0.09-0.12] (58.5%) | 0.55 [0.53-0.56] | 0.03 [0.00-0.04] (5.6%) | 0.38 [0.36-0.41] | 0.10 [0.08-0.13] (34.4%) |
| Scaphoid | 0.41 [0.37-0.41] | 0.14 [0.13-0.16] (55.6%) | 0.58 [0.54-0.65] | 0.01 [0.01-0.01] (0.3%) | 0.48 [0.47-0.49] | 0.13 [0.13-0.14] (36.2%) |
| Trapezium | 0.37 [0.33-0.40] | 0.12 [0.10-0.15] (46.2%) | 0.65 [0.60-0.65] | 0.03 [0.02-0.04] (5.6%) | 0.40 [0.39-0.46] | 0.09 [0.08-0.12] (29.6%) |
| Trapezoid | 0.41 [0.34-0.41] | 0.14 [0.09-0.14] (48.0%) | 0.59 [0.58-0.61] | 0.01 [0.00-0.01] (1.2%) | 0.44 [0.41-0.47] | 0.10 [0.10-0.11] (31.0%) |
| Triquetrum | 0.40 [0.33-0.40] | 0.13 [0.11-0.17] (49.8%) | 0.60 [0.58-0.60] | 0.03 [0.02-0.03] (5.6%) | 0.42 [0.39-0.45] | 0.11 [0.09-0.13] (30.9%) |
| All | 0.38 [0.33-0.41] | 0.13 [0.10-0.16] (52.2%) | 0.59 [0.57-0.64] | 0.02 [0.01-0.03] (2.7%) | 0.45 [0.40-0.48] | 0.12 [0.09-0.14] (33.8%) |
| *Adaptive Threshold* |  |  |  |  |  |  |
| Capitate | 0.32 [0.29-0.32] | 0.03 [0.02-0.04] (13.4%) | 0.56 [0.55-0.56] | 0.01 [0.01-0.01] (0.7%) | 0.40 [0.35-0.40] | 0.01 [0.01-0.02] (4.1%) |
| Hamate | 0.31 [0.28-0.31] | 0.05 [0.04-0.05] (20.3%) | 0.58 [0.57-0.64] | 0.02 [0.01-0.03] (-1.1%) | 0.37 [0.32-0.37] | 0.02 [0.02-0.03] (7.0%) |
| Lunate | 0.30 [0.29-0.31] | 0.05 [0.05-0.05] (22.1%) | 0.55 [0.52-0.60] | 0.02 [0.02-0.02] (-3.4%) | 0.38 [0.37-0.38] | 0.04 [0.03-0.05] (11.9%) |
| Pisiform | 0.27 [0.25-0.27] | 0.06 [0.06-0.07] (29.8%) | 0.54 [0.51-0.54] | 0.02 [0.02-0.02] (2.3%) | 0.33 [0.32-0.35] | 0.05 [0.03-0.06] (17.2%) |
| Scaphoid | 0.30 [0.29-0.30] | 0.04 [0.04-0.06] (17.3%) | 0.55 [0.53-0.61] | 0.02 [0.02-0.03] (-4.4%) | 0.38 [0.37-0.39] | 0.03 [0.02-0.05] (7.9%) |
| Trapezium | 0.29 [0.28-0.30] | 0.05 [0.04-0.05] (19.8%) | 0.61 [0.58-0.63] | 0.01 [0.01-0.02] (-0.4%) | 0.34 [0.34-0.36] | 0.02 [0.02-0.03] (7.5%) |
| Trapezoid | 0.30 [0.29-0.31] | 0.04 [0.04-0.04] (16.3%) | 0.58 [0.57-0.59] | 0.01 [0.01-0.03] (-2.1%) | 0.36 [0.35-0.37] | 0.03 [0.02-0.03] (8.5%) |
| Triquetrum | 0.30 [0.28-0.30] | 0.05 [0.05-0.06] (24.0%) | 0.57 [0.57-0.57] | 0.01 [0.01-0.02] (2.5%) | 0.34 [0.34-0.36] | 0.03 [0.03-0.04] (10.4%) |
| All | 0.30 [0.28-0.31] | 0.05 [0.04-0.06] (19.6%) | 0.57 [0.55-0.61] | 0.02 [0.01-0.02] (-1.8%) | 0.37 [0.34-0.38] | 0.03 [0.02-0.04] (8.5%) |

***
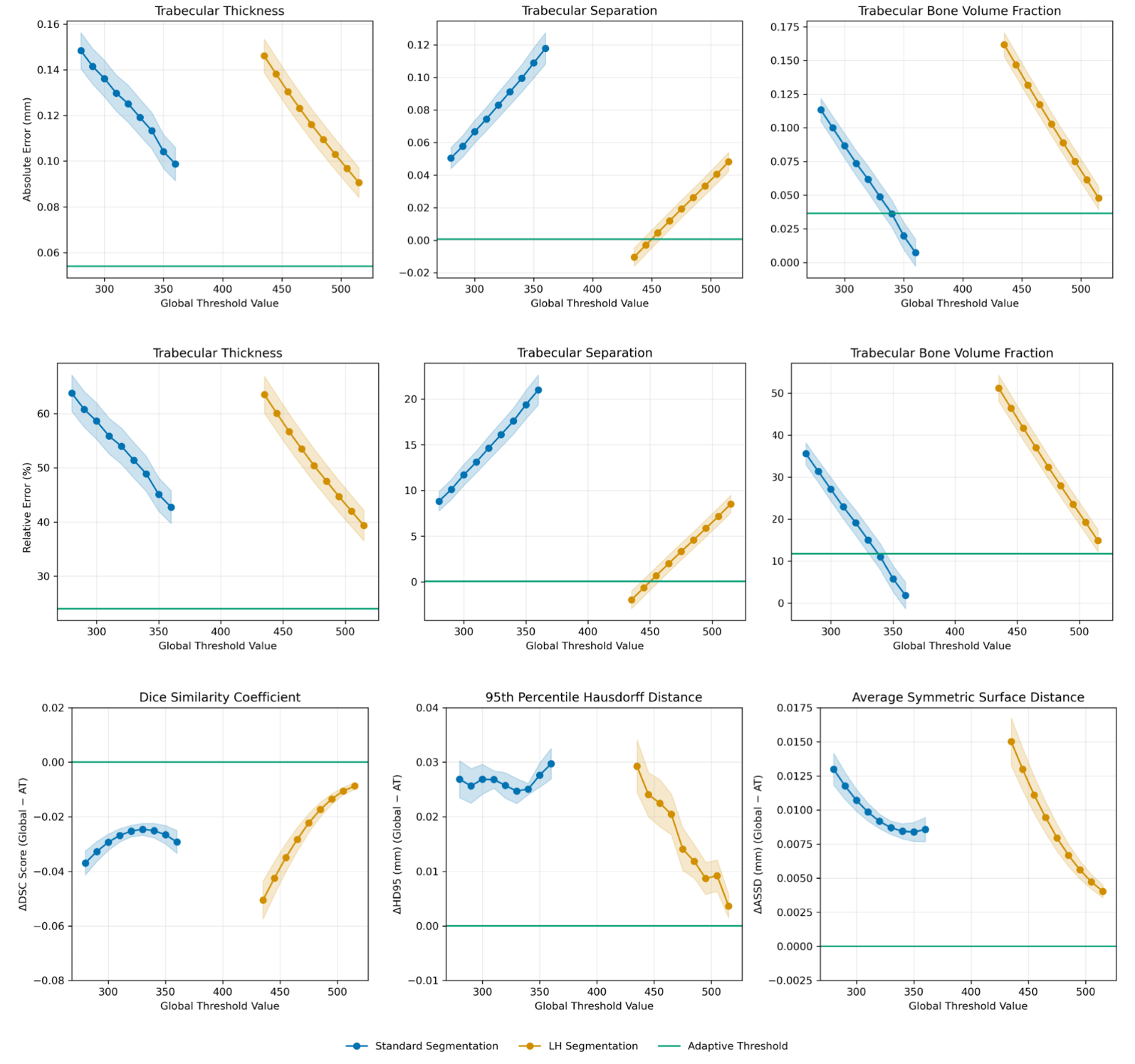
Supplemental Figure 1:*** *Results of the sensitivity analysis investigating the effects of global threshold value on trabecular bone microarchitecture and spatial overlap accuracy relative to µCT. Global thresholds for the standard method were varied from 280-360 mg HA/cm3, in increments of 10 mg HA/cm3, and from 435-515 per mille for the Laplace-Hamming method, in increments of 10 per mille. Absolute (top row) and relative (middle row) error in trabecular thickness, separation, and bone volume fraction were computed for each segmentation method, at each global threshold value. Difference in spatial overlap metrics, including Dice similarity coefficient (DSC), 95^th^ percentile Hausdorff distance (HD95), and average symmetric surface distance (ASSD) were also computed at each global threshold value (bottom row). Since the adaptive local threshold method does not rely on a fixed global threshold, results from the adaptive local threshold method are shown with a solid green line.*
